# Slab rollback orogeny in the Alps and evolution of the Swiss Molasse basin

**DOI:** 10.1038/ncomms9605

**Published:** 2015-10-16

**Authors:** Fritz Schlunegger, Edi Kissling

**Affiliations:** 1 Institute of Geological Sciences, University of Bern, Baltzerstrasse 1+3, CH-3012 Bern, Switzerland; 2 Institute of Geophysics, ETH Zürich, Sonneggstrasse 5, CH-8092 Zürich, Switzerland

## Abstract

The stratigraphies of foreland basins have been related to orogeny, where continent–continent collision causes the construction of topography and the downwarping of the foreland plate. These mechanisms have been inferred for the Molasse basin, stretching along the northern margin of the European Alps. Continuous flexural bending of the subducting European lithosphere as a consequence of topographic loads alone would imply that the Alpine topography would have increased at least between 30 Ma and *ca*. 5–10 Ma when the basin accumulated the erosional detritus. This, however, is neither consistent with observations nor with isostatic mass balancing models because paleoaltimetry estimates suggest that the topography has not increased since 20 Ma. Here we show that a rollback mechanism for the European plate is capable of explaining the construction of thick sedimentary successions in the Molasse foreland basin where the extra slab load has maintained the Alpine surface at low, but constant, elevations.

## Abstract (web summary)

The stratigraphy of the Molasse basin has been related to the convergent regime, but aspects do not fit with observations or isostatic models. Here, the authors show that rollback of the European plate may have caused the thick sedimentary successions in the foreland basin, independent of plate convergence mechanisms.

## Graphical abstract

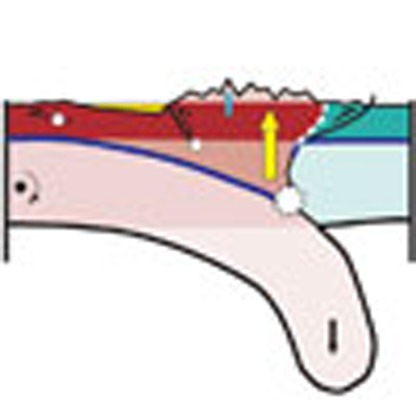

The peripheral Oligo/Miocene Molasse Basin, situated on the subducting European plate on the northern side of the Alpine orogen, has been considered as one of the most classical examples for a foreland basin system^1^,^2^. In general, peripheral foreland basins develop on a continental lithosphere adjacent to evolving mountain belts at the boundary between two convergent continental plates^3^,^4^,^5^,^6^. In these basins, the stratigraphic evolution is closely linked to the plate convergence rates and the related crustal thickening in the adjacent mountain belt^3^,^4^,^5^,^6^ that resides on the upper plate. These mechanisms drive the subsidence of the foreland basin through subduction slab load and topographic load forces, which downwarp the foreland plate and create a sedimentary trough^3^. Orogenic processes also condition the redistribution of topographic loads to the adjacent basin through erosion and redeposition^3^,^4^,^5^,^6^.

In the past years, detailed magnetopolarity stratigraphies established for the Oligo/Miocene deposits of the Molasse Basin^7^,^8^,^9^,^10^,^11^ have yielded a chronological framework with a temporal resolution of <500 Ka. These data sets have provided the basis to disclose the causal relationships between the construction of orogenic loads in the Alps, and the flexural response of the basin^9^,^10^,^12^,^13^. These processes, in turn, have been linked to the convergence and the subsequent collision between the Adriatic and European continental plates, leading to the formation of the Alps through thrusting, crustal stacking and erosional recycling^14^,^15^. While these relationships are clear, efforts to explain the large-scale subsidence pattern of basin and the accumulation of km-thick megafan conglomerates through the build up of topographic loads alone have been contested^15^.

Here we present an alternative explanation where this foreland basin evolved in response to slab rollback processes^16^,^17^, where subduction slab load^3^ and crustal root buoyancy forces have been the major driving mechanisms. We proceed by synthesizing, along a representative transect through the Central Alps of Switzerland, published information about the history of crustal accretion of the Alps, the development of the adjacent Molasse foreland basin to the north and the evolution of the surface topography and sediment discharge between *ca.* 35 Ma and the present. We use these data to propose a mechanism where tectonic processes related to slab rollback orogeny conditioned the subduction processes, the formation of accommodation space in the Molasse Basin and the crustal accretion in the adjacent Alps. We suggest that a slab rollback orogeny scenario for the Alps and the Molasse Basin is capable of reconciling previously conflicting stratigraphic, palaeoaltimetry and also tectonic observations.

## Results

### Tectonic architecture of the Alps

**Figure 1:**
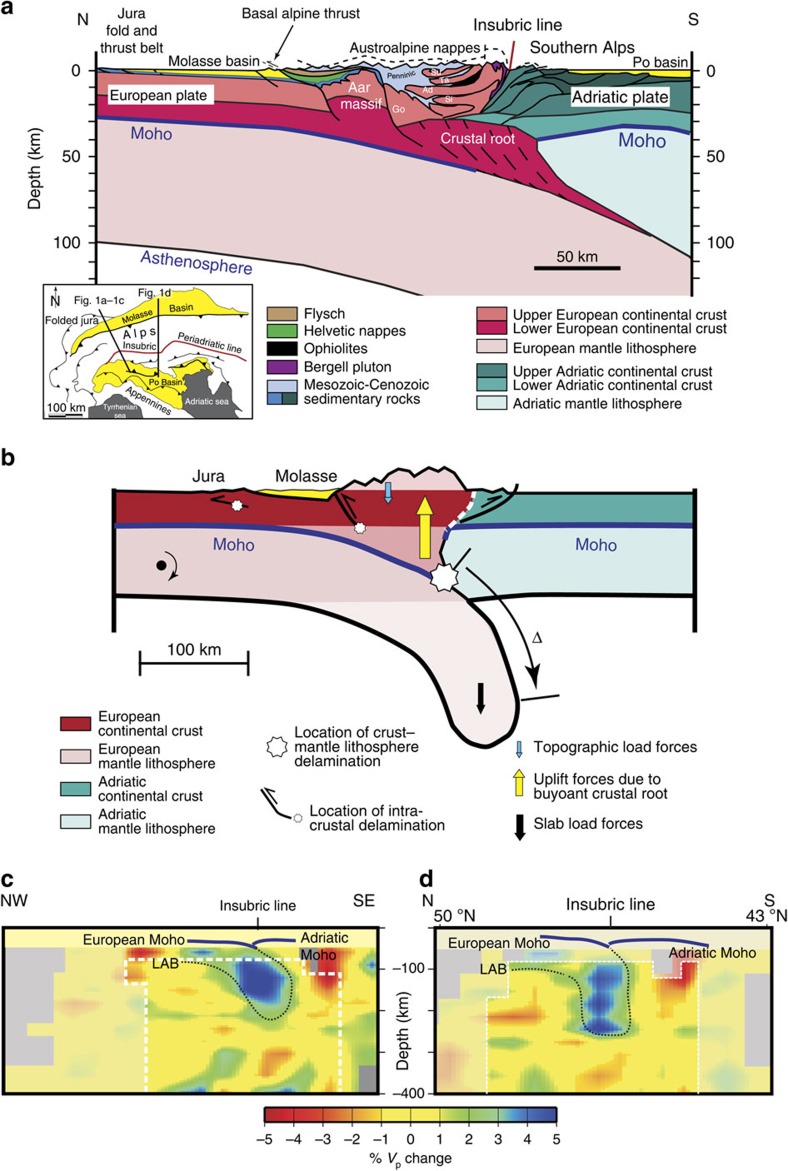
The Alps and the Molasse basin. (**a**) Inset map illustrating the Alps and its foreland basin along with the sections that are analysed in this paper, and tectonic section across the Central Alps, which has been updated from Figure 6 of Fry *et al.*^20^ on the basis of the results of seismic reflection and refraction sections^14^,^18^ and high-resolution teleseismic surveys^18^,^19^,^20^,^21^. The crystalline substratum of the Penninic sedimentary units comprises the following nappes: Go=Gotthard massif, Si=Simano; Ad=Adula, Ta=Tambo, Su=Suretta. (**b**) Conceptual sketch figure for the geophysical architecture of the Swiss Alps illustrating the following: isostatic forces operating on the orogeny (topographic load forces, slab load forces and crustal root buoyancy forces), sites of crustal delamination and length Δ of subducted European lithospheric mantle, while lower and upper crusts have been delaminated and accreted into the Alps (Fig. 1a). (**c**,**d**) Teleseismic tomography cross-sections^19^ illustrating European slab geometry beneath the central Alps. For location of profiles see inset map. The figure shows lateral variations of P-wave velocity *V*_p_ beneath the Moho. These variations are illustrated as changes relative to a one-dimensional velocity model. LAB=Lithophere Astenosphere Boundary.

Reconstructions of the current tectonic architecture of the Alps^14^ are based on sedimentary archives, petrographic and structural mapping and geophysical information^18^. Recently, teleseismic tomography imaging disclosed details of the deepest structure of the Central Alps (Fig. 1a), where a southward dipping lithospheric slab of >160 km length is attached to the European plate^19^ (Fig. 1b–d). In their central part, above this lithospheric slab and situated almost exclusively on the subducting European plate, the Alps (Fig. 1a) are made up of a crustal root^20^, and a doubly vergent nappe stack with a crystalline core^14^. Extensive geophysical surveys and deep crustal mapping revealed that the material of the crustal root has been derived from the subducting European plate^18^,^21^. The Alpine nappes are grouped according to their paleogeographic positions during the Mesozoic phase of plate tectonic spreading. On the northern side, the Helvetic units, which straddle the basal Alpine thrust, represent the former shelf areas of the European passive margin^14^, while the Penninic units constitute the sedimentary realm in the distal stretched part of the European continent^22^. During subsequent convergence, the sedimentary units of these domains were displaced farther to the north than their crystalline substrata that are preserved as a nappe stack in the centre of the orogen^14^ (Fig. 1a). The Austroalpine and Southalpine nappes comprise basement and overlying sedimentary rocks that were part of the Adriatic continental plate^14^. The Insubric line, situated near the southern limits of the Swiss Alps, operated as backstop between the Late Oligocene and the Early Miocene when a large portion of shortening was accommodated by backthrusting and right-lateral slip^14^. At present, both margins of the Alps are delineated by fold and thrust belts that are referred to as the Jura Mountains and the Southern Alps on the northern and southern sides, respectively.

### Evolution of the Alps

Reconstructions of orogenic processes leading to the current architecture of the Alps are mainly based on a combination of thermobarometric investigations^23^,^24^,^25^,^26^, analyses of structural data sets including the bedrock's fabric and cross-cutting relationships thereof^14^. We additionally considered the conservation of mass in our reconstructions, which is adapted here through crustal mass and line balancing. In the Alps, today's main tectonic architecture is the consequence of the subduction–collision history, which started with the subduction of the European oceanic lithosphere beneath the Adritatic continental plate and the closure of the Tethys Ocean during the Late Cretaceous^14^. Closure of this ocean was accomplished by the subduction of the Penninic realm beneath the Austroalpine units that represented the northern margin of the Adriatic continental plate, and that also formed the orogenic lid during subduction processes^14^. During that time, the thrust underlying the Austroalpine nappes (Fig. 1a) formed the boundary between the Adriatic and European plates^14^.

At *ca.* 35 Ma, ocean–continent subduction was superseded by continent–continent collision, when the buoyant European continental lithosphere with a lower flexural rigidity than the previously consumed oceanic lithosphere^14^ started to enter the subduction channel. These circumstances created extensional forces within the slab, driven by differences in buoyancy forces and flexural strengths between the subducted, dense and relatively stiff oceanic lithosphere and the continental lithosphere. The differential forces at work resulted in oceanic lithosphere slab break off, the heating of the overriding lithosphere by the upwelling asthenosphere and the generation of magmas, which intruded through the Alpine nappe stack between 30 and 32 Ma (ref. 23) to form the Bergell granites (Fig. 1a). Slab break off was also accompanied by rapid rock uplift and orogen-parallel extension near the southern limits of the Swiss Alps. This was accomplished through backthrusting along the Insubric Line^14^ and slip along low-angle detachment faults^24^, resulting in vertically directed extrusion of deep-seated rocks and widespread exposure of crystalline rocks on the orogen surface^14^,^25^,^26^. Between *ca.* 30 and 20 Ma, the Insubric Line and the basal Alpine thrust represented the Alpine margins on the southern and northern sides, respectively^14^,^27^. In addition, since *ca.* 30 Ma, the Insubric Line has taken the role as a boundary between the European and Adriatic continental plates^14^. Rapid exhumation in response to slab break off was completed at 20 Ma at the latest, when the orogen front continued to involve previously deposited Molasse sediments^2^,^9^,^10^.

Between collision times and 20 Ma, the orogen as a whole migrated *ca.* 80–100 km farther north^14^,^15^. After 20 Ma, on the southern side of the Alps, the deformation front shifted from the plate boundary (Insubric Line) some 50 km to the Adriatic plate^27^, thereby forming the Southern Alpine nappes (Fig. 1a). Finally, at *ca.* 10 Ma, rock uplift and folding involved the Jura fold and thrust belt^28^,^29^ on the northern margin of the Molasse Basin, which resulted in a widening of the convergence belt to >200 km.

At deeper crustal levels, the subduction of the European continental plate has occurred in concert with delamination of buoyant lower crustal material from the downgoing plate and the stacking of the crustal root^20^, while the lithospheric mantle was subducted (Fig. 1a). Ongoing collision caused the site of crustal delamination to shift farther to the north and to shallower crustal levels, resulting in the uplift of the Aar massif along steep faults since 20 Ma (ref. 14), and finally in thick-skinned faulting beneath the Jura fold and thrust belt^30^ (Fig. 1a,b).

Reconstructions of the chronology of thrusting and faulting following slab break off together with crustal mass and line balancing yielded a total of *ca.* 160 km of crustal shortening^14^, which corresponds to the length of the subducted lithospheric mantle that is still attached to the European continental plate^18^ (Fig. 1).

### Buoyancy and subduction slab load forces beneath the Alps

Strong buoyant forces at work have been inferred from negative Bouguer gravity anomalies of <−180 mGal in the Central Alps, leading to the notion that the Alpine root is overcompensated by many kilometres^31^,^32^,^33^. Accordingly, slab loads exerted by the >160-km-long subducted European lithospheric mantle in addition to surface topographic loads (Fig. 1b) are required to balance the buoyancy forces exerted by the crustal root and to maintain the Alpine topography at relatively moderate mean elevations of 1,000–2,500 m above sea level. This can be exemplified using basic concepts of isostatic compensation to applied loads (Fig. 1b). In the simplest case of Airy–Heiskanen isostasy where the local accommodation of crustal thicknesses conditions the topographic heights, the buoyancy of a *ca.* 30-km-thick crustal root^34^ would maintain a mountain belt twice as high as the current Alps if these forces were not balanced by a downward directed load force. In the more complex case of Vening Meinesz isostasy, where isostatic accommodation of applied loads is accomplished across larger scales through the lithosphere, the mean elevation of such a mountain belt would even be higher.

The vertical load force and resulting bending moment caused by the subducted European lithospheric mantle also explains the seismicity beneath the Molasse Basin, where focal mechanisms of 20–30-km-deep earthquakes point to the occurrence of extensional forces at work within the crust^35^. Accordingly, the combination of negative Bouguer gravity anomalies in the Central Alps^31^ and focal mechanism solutions of earthquakes beneath the Molasse Basin^35^ calls for a plate tectonic regime that is characterized by an extensional rather than a compressional driving force, which we relate here to the vertical load forces generated through rollback mechanisms of the subducted lithospheric mantle.

### Stratigraphy and evolution of the Molasse Basin

**Figure 2:**
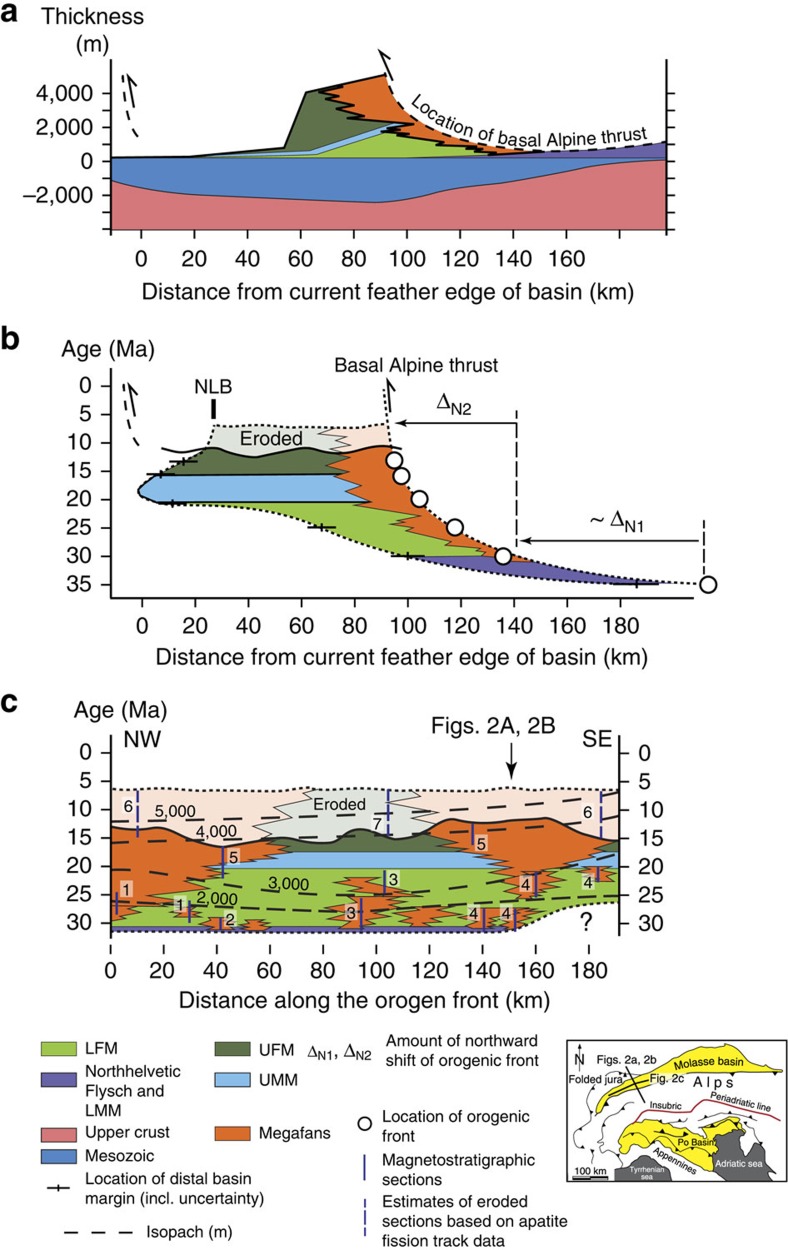
Stratigraphic data from the Molasse basin. (**a**) Cumulative thicknesses of Molasse units across a S–N section^36^ and locations relative to the orogen front where sediment accumulation has occurred. Inset Figure next to the legend illustrates the orientation of the section, which is identical as Fig. 1c. (**b**) Chronologic (Wheeler) diagram for Molasse units across the basin. The figure has been modified from Sinclair *et al.*^37^ and expanded for post 15–12-Ma-old units, which have been eroded and recycled after 5–6 Ma (ref 41, 42). NLB=Northern Limit of Basin. For orientation of section see figure next to the legend. (**c**) Chronologic (Wheeler) diagram^43^ for Molasse units for a section that parallels the proximal basin border (see inset figure next to the legend for orientation of section). The numbers 1 (ref. 7), 2 (ref. 8), 3 (ref. 9), 4 (ref. 10) and 5 (ref. 11) correspond to the sources of the magnetostratigraphic data. Sections labelled with 6 (ref. 41) and 7 (ref. 42) have been analysed for the time and the amplitude of basin erosion on the basis of fission track data established for detrital apatites encountered in drillings. Isopachs for non-decompacted Molasse sediments^15^ are based on thicknesses of magnetostratigraphic sections. The inset Figure next to the legend illustrates the Alps, the Molasse and Po Basins together with the orientation of the sections.

The Oligo/Miocene deposits of the Molasse Basin, situated north of the Alps, have been categorized into five stratigraphic groups^1^,^2^,^12^,^13^,^36^,^37^,^38^ that represent two progradational and regressive megasequences (Fig. 2). They record two periods during which sediment discharge into the basin increased more rapidly than accommodation space in the basin was formed^38^. The earliest of these megasequences, represented by the pre-30-Ma-old North Helvetic Flysch, the Lower Marine Molasse and the 30–25-Ma-old Lower Freshwater Molasse groups (LFM), chronicles the transition from the early underfilled Flysch to the overfilled Molasse stage of basin evolution^38^. The second megasequence formed during filled to overfilled conditions^38^. The corresponding units are the 20–17-Ma-old Upper Marine Molasse (UMM) and post-17-Ma-old Upper Freshwater Molasse groups (UFM).

At the proximal basin border, palinspastic restorations revealed that the orogen front shifted 90–110 km farther north between 35 Ma and the present (sum of Δ_N1_ and Δ_N2_ on Fig. 2b). At the proximal basin border, propagation rates of the Alpine front slowed down from initially >8 mm per year prior to 30 Ma to <2 mm per year between 30 and 10 Ma, and finally to <0.5 mm per year thereafter (Fig. 2b). On the northern side of the Molasse Basin, northward expansion of its distal margin occurred in concert with the northward migration of the orogen prior to *ca.* 30 Ma (refs 36, 37). Downwarping of the foreland plate and accommodation space formation were also associated with normal faulting^2^. The latter is particularly documented at 46°56′ N and 8°07′ E where mapping shows the occurrence of m^3^-large olistolites adjacent to a normal fault. Propagation rates of the distal basin border were faster than those of the orogenic front during LFM times when the basin was overfilled (Fig. 2b). This can be explained by the large sedimentary fluxes^39^,^40^, which is related to the rapid build up of the Alpine topography in response to oceanic lithosphere slab break off^40^, thereby accommodating an isostatic balance between the thick buoyant crustal root and the vertical loads exerted by the mantle slab and surface topography (Fig. 1b).

Accommodation space was formed through subsidence of the foreland plate^1^,^2^,^10^,^12^,^13^,^15^,^36^,^37^. At the proximal basin margin, the amplitude of the deflection increased from *ca.* 2,000 m prior to 25 Ma to 3,000 m at *ca.* 20 Ma, and finally to 3,000–5,000 m during UFM times (Fig. 2a,c). Likewise, the basin's width increased from 40 to 50 km between 30 and 25 Ma, and then to 100 km at 20 Ma, after which the basin narrowed to 60 km (Fig. 2b). The corresponding dip angles of the foreland plate, averaged across the entire basin, measured between 1.7–2.4° during LFM and UMM times and 2.9–4.8° thereafter. Sediment accumulation continued until the Miocene–Pliocene boundary, when a large-scale phase of uplift inverted the basin (Fig. 2), resulting in erosion and recycling of previously deposited foreland basin material during the past 5–6 Ma (refs 41, 42), thereby driving high sediment discharges from recycling of Molasse deposits^39^.

### Evolution of Alpine topography

**Figure 3:**
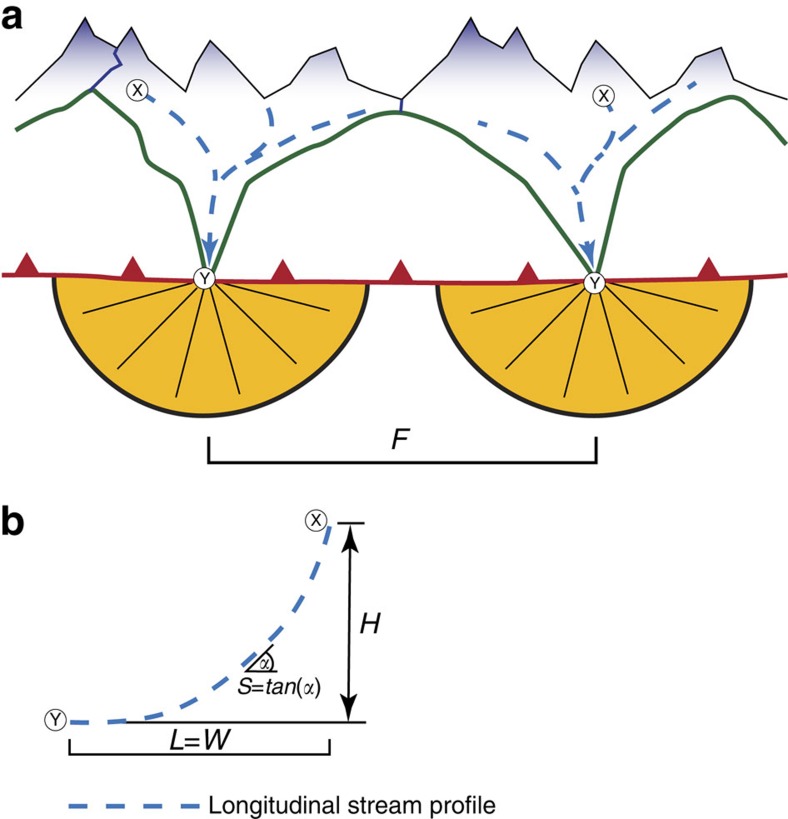
Drainage basin geometry. (**a**) The spacing *F* between fan depocentres depends on the cross-sectional width *W* at which the streams have eroded into the orogen^45^. This variable, in turn, is related to the length of the stream *L* within the mountain belt. (**b**) longitudinal stream profile, showing variables used in this paper including stream length *L*, channel gradients *S* and total basin relief *H*.

**Figure 4:**
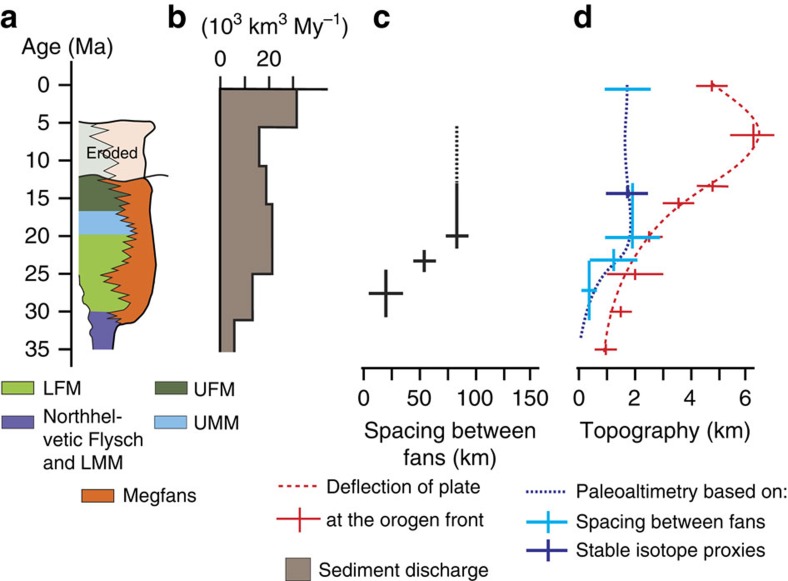
Stratigraphy of the Molasse Basin and morphometric properties of the Alps. (**a**) Stratigraphic chart of the Molasse Basin^12^, illustrated here for a synthetic section from the proximal border to the distal part of the basin. (**b**) Pattern and rates of sediment discharge for the Central Alps, which is based on the sediment budgets of material that has been deposited in circum-Alpine basins and that has been derived from the Central Alps^39^. (**c**) Spacing *F* between fans, taken from Fig. 2c. (**d**) Paleoaltimetry estimates (dashed blue line) and foreland basin deflection at proximal basin border (dashed red line) for various times. Error bars are s.e.m. (stable isotope proxies) or correspond to minimum and maximum values that resulted from the applications of equations (1, 2, 3).

Stratigraphic analyses paired with mapping reveals the occurrence of seven to eight megafans that were established at the proximal basin border prior to 25 Ma (Fig. 2c). It is possible, however, that the number of small fans would even be larger as a portion of LFM deposits is buried beneath the Alpine nappes. These fans are represented by well-sorted, clast-supported conglomerates^8^,^9^,^10^, where individual stratigraphic units are between 5 and 10 km wide^43^ and predominantly comprise sedimentary clasts^9^,^10^. At 25 Ma, the seven to eight depocentres had merged to a total of four megafans with cross-sectional widths >10 km, and then finally to two systems thereafter, when the fan widths expanded to nearly 30 km (Fig. 2c). Likewise, the spacing *F* between the individual fans (Fig. 3) widened from 10–30 to 50–70 km between 31 and 25 Ma and finally to 80–100 km since 22–20 Ma, after which the spacing remained at a constant value of *ca.* 100 km (Figs 2c and 4c). The corresponding stream lengths *W* (Fig. 3) increased from 20–60 km to *ca.* 100–140 km between 31 and 25 Ma, and then finally to >150 km thereafter. This change in the dispersal geometry was also associated with shifts in the clast suites of the conglomerates, where the relative contribution of crystalline clasts increased from <30% prior to 27 Ma to >60% after 25 Ma, particularly for those streams that had their sources in the core of the Alps^9^,^12^.

The change in the deposition and dispersion geometry was most likely accomplished by headward retreat of the erosional front in the Alps^43^, starting with the incision into the frontal sedimentary nappes at *ca.* 30 Ma, and reaching the crystalline core between 25 and 22 Ma at the latest. The inferred headward shift of the erosional front is consistent with the larger contribution of crystalline constituents in the conglomerates^9^,^12^. This mechanism also resulted in an increase in the supply rate of sediment to the Swiss Molasse basin from initially <*ca.* 5 × 10^3 ^km^3 ^My^−1^ at 30 Ma to nearly 20 × 10^3 ^km^3 ^My^−1^ after 25 Ma (ref. 39; Fig. 4b).

Headward erosion of the Alpine streams was most likely accomplished through drainage divide migration and cannibalization^43^ of drainage basins, where more powerful rivers captured the headwaters of less erosive neighbouring streams^44^. We use these mechanisms to explain the observed decrease in the number of fan depocentres from initially seven to eight prior to 27 Ma to *ca.* four at 25 Ma, and finally to two thereafter (Fig. 2c). Headward retreat paired with stream cannibalization also resulted in an up to five times larger sediment discharge per dispersal system and thus in the observed enlargement of the fan widths if the total sediment flux is equally distributed among the drainage basins^43^.

We use the spacing (Figs 3 and 4c) and cross-sectional widths of alluvial megafan conglomerates^43^,^45^, together with steepness values of 100 and 300 m typical for mountainous streams^46^,^47^,^48^,^49^,^50^ to estimate the range of possible palaeo-elevations for the Alpine drainage divide (Figs 3 and 4d), which likewise corresponds to the total fluvial relief *H*. Depending on assignments for the steepness and Hack's variables^43^,^51^, the relative elevation of the drainage divides increased from 300±200 to 1,200±900 m between 31 and 25 Ma, and then to 1,900±1,000 m after 22 Ma. The latter value corresponds to the currently measured total fluvial relief between *ca.* 1,000 and 2,500 m (Fig. 4d). These estimates for the post-20-Ma palaeo-elevations are consistent with independent constraints that are based on a large range of evidence^52^. In addition, *δ*D values established for Middle Miocene caliche nodules in the Molasse Basin and 14.5-Ma-old micas in Alpine fault gouges have been used to infer a total fluvial relief of 2,300±500 m during UFM times^53^. The uncertainties on these estimates are large, but the general trend towards an increasing total fluvial relief until *ca.* 20 Ma, followed by a prolonged period of time during which this variable has remained nearly constant will not change.

## Discussion

Continent–continent collision between the Adriatic and European plates and subduction of the European foreland has been considered as the driving mechanism for the evolution of the Alps and the Molasse basin^1^,^2^,^9^,^10^,^12^,^13^,^15^,^36^,^37^,^38^. According to these views, the basin subsided as topographic loads were constructed, which also conditioned the high erosional flux in the Alps. However, topographic loads alone cannot explain the formation of accommodation space in the Molasse basin^15^ for two lines of arguments. First, seismically determined Moho maps^54^ and the negative Bouguer gravity of <−180 mGal in the Central Alps^31^ document the presence of a buoyant crustal root^20^,^33^, which requires a vertically oriented slab load force in addition to surface topography to balance the lifting force^18^ (Fig. 1b). Second, the continuous augmentation of the basin's deflection during UFM times (Fig. 4d) would ask for an increase in the orogeny's altimetry, if most of the basin's deflection were accomplished through topographic loads^3^,^4^,^5^,^6^. An increasing altimetry, in turn, would be associated with a larger total relief of mountainous streams and steeper channels. These variables, in turn, would be linked with a larger erosional flux as channel gradients, basin relief and surface erosion rates are positively correlated^49^,^55^ where streams directly discharge into the foreland, as was the case for the Alps during most of their evolution^43^. While an increasing relief, paired with headward retreat of the erosional front, can be invoked to explain the Late Oligocene increase in sediment supply to the Molasse Basin^43^, such a scenario is not consistent with observations after *ca.* 20 Ma as both variables remained constant since then (Fig. 4d), or at least they did not increase. Alternatively, a scenario where horizontal forces cause a downward dragging of the foreland plate by slip along the subduction boundary offers a valuable explanation for the decoupling between basin depth and topographic loads^56^, particularly during UFM times (Fig. 4). However, such a scenario would be associated with the occurrence of compressional forces within the foreland plate. This is not in agreement with observations in the Molasse Basin where focal mechanisms of seismic events imply the occurrence of extensional forces at work, at least at present^35^,^57^. Likewise, horizontal forces at the plate boundary interface are not capable of balancing the vertical buoyancy forces that have their origin in the crustal root at least since Oligocene times^18^.

Rollback orogeny, driven by the gravitational pull of the European slab, provides a viable mechanism to explain the increasing deflection of the foreland in the absence of larger topographic forcing (Fig. 4). It also offers the conditions that promote the accretion of crustal material from the subducting plate, and it agrees with the geologic record that the subducting European plate did not move south while the overriding Adriatic plate shifted north^58^. Such a model is presented in the next section.

**Figure 5:**
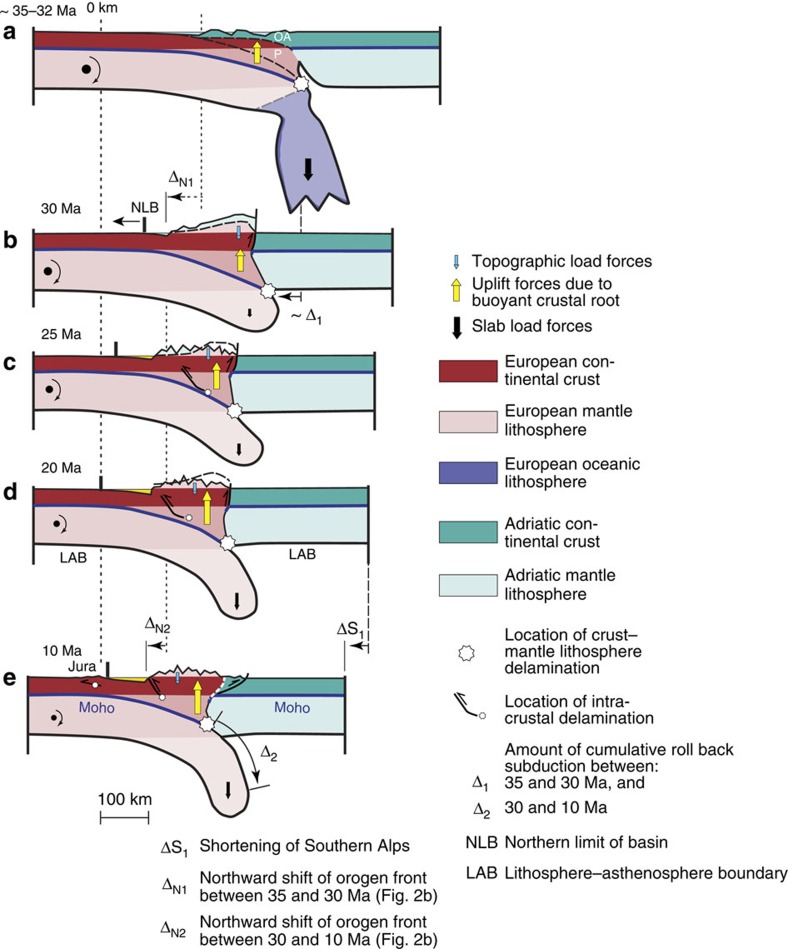
Flexural evolution of Alpine orogeny at different times. (**a**) Necking and oceanic slab break off (∼35–32 Ma). Initiation of subduction of continental European plate with lower rigidity marked the onset of collision and created extensional forces within the lower plate, resulting in necking and slab breakoff at the interface between the continental and oceanic European lithospheres. OA=Australpine nappes, forming orogenic lid; P=Penninic nappes (Fig. 1a). (**b**) End of Lower Marine Molasse deposition after oceanic slab break off (30 Ma). The removal of loads exerted by the dense oceanic lithosphere resulted in a phase of rapid surface uplift and the establishment of a high-elevated Alpine plateau, which was poorly dissected at that time. Vertical load forces exerted by the subducted continental lithosphere and upward buoyant forces caused by the crustal root exerted tensile stresses at the Moho and in the European lithosphere, with the result that mantle delamination and slab rollback proceeded further. Δ_N1_=Northward shift of Basal Alpine Thrust (Fig. 2b). (**c**) Ongoing mantle slab rollback, fast surface erosion and formation of Alpine landscape, and deposition of LFM (25 Ma). (**d**) Mantle rollback slowed down, marine transgression and deposition of the UMM (20 Ma). Heating of the subducted slab decreased the flexural strength of the foreland plate, with the result that the curvature of the subducted plate increased and reached deeper levels in the asthenosphere. This mechanism localized and increased the tensile stresses at the Moho, but also within the crust, with the consequence that the site of intracrustal delamination reached shallower crustal levels. This resulted in the accretion of the Aar massif (Fig. 1), and finally in the uplift of crustal blocks along steep faults beneath the Jura fold and thrust belt (Fig. 1a). (**e**) Deposition of UFM and block uplift beneath the Jura (10 Ma). This Figure additionally sketches the complexity of the plate boundary evolution during the past 35 Ma.

The removal of the oceanic lithospheric slab load during slab break off significantly reduced the slab load, which in turn relieved some of the forces that were holding the existing load farther down. The result was a shift of the surface elevation, yielding a larger topographic load (Fig. 1b) to maintain the isostatic balance with the buoyant crustal root (Fig. 5a). We use these mechanisms to explain the rise of the Alpine topography to the modern elevations within a few My during Late Oligocene times (Fig. 4d). The result was an increase in the sediment discharge during the Late Oligocene^39^ (Fig. 4b), which terminated the underfilled Flysch stage at 30 Ma (ref. 40). During the overfilled Molasse stage between 30 and 10 Ma (Fig. 5b–e), the increase in the basin's deflection at constant topographic loads (Fig. 4d), the deceleration in the northward propagation of the Alpine front (Figs 2b and 5) and the shift of intracrustal and/or crust–mantle delamination, or decoupling^17^, to shallower levels (Fig. 5) call again for an increase in the relative importance of the slab load force. This was most likely accomplished through ongoing slab rollback, where the velocity was conditioned by the delamination of the crust from the mantle lithosphere as a consequence of divergent forces between the buoyant crust and the mantle slab load. The divergent forces at work resulted in tensile stresses at the crust–mantle boundary but also within the crust, thereby promoting intracrustal delamination and accretion of crustal material along steep faults. In addition, these processes most likely created a positive feedback where delamination of buoyant material from the subducting plate augments the relative importance of the vertical slab load, thereby promoting further downwarping of the plate. We consider that such a mechanism yielded a foreland basin where the subsidence increased while the flexural wavelength and the northern basin limits remained stationary, as has been the case in the Molasse Basin since *ca.* 20 Ma (Fig. 5). Furthermore, this mechanism not only localized and increased the tensile stresses at the Moho but also within the crust, with the consequence that the site of intracrustal delamination reached shallower crustal levels.

In addition, heating of the subducted slab most likely decreased the flexural strength of the foreland plate, with the result that the curvature of the subducted plate increased, thereby reaching deeper levels in the asthenosphere similar to a thermoelastic relaxation of a continental plate to applied loads^59^. Along a N–S transect at 10°E longitude (Fig. 1d) the mantle lithosphere slab, which is attached to the European plate, hangs in a vertical position beneath the core of the Alps, while along the NW–SE transect (Fig. 1a–c) the slab dips with only ∼60°. This documents the occurrence of some remaining flexural rigidity in the European lithosphere beneath the western part of the Molasse Basin (Fig. 1a). In the context of our evolutionary reconstructions (Fig. 5), a reduction of elasticity would result in a slow down of the migration of the crust–mantle delamination location. Eventually, thermomechanical loss of elasticity in the mantle lithosphere (Fig. 1d) will bring the migration processes and thus slab rollback to a halt.

Slab rollback orogeny, the stacking of the crustal root and concepts of rock mechanics applied to mountain belts provide a mechanism to explain the post-20-Ma widening of the Alps. In particular, the crustal buoyancy forces at work in the deep crustal root in combination with decreasing topographic loads towards the northern Alpine front^2^,^14^,^15^ have promoted continuous lateral extrusion of European upper and middle crust towards the NW as, for example, documented by the uplift of the Aar massif after 20 Ma (ref. 14). The result is crustal shortening along the northern margin of the Alps contemporary with orogen-parallel extension within the crystalline core of the Penninc nappes stack north of the Insubric Line (for example, faulting along the Simplon detachment fault^24^). This crustal material flux led to the continuous northwestward migration of the northern orogen front in conjunction with a net increase in orogen width and thrusting of proximal Molasse deposits until *ca.* 15–13 Ma (refs 2, 10), after which the site of main shortening shifted to the Jura thrust belt at 10 Ma at the latest^28^,^29^. Furthermore, besides orogen-parallel extension along shallow dipping normal faults^24^, continuous vertically directed crustal flux led to the southward shift of the Southern Alpine margin at *ca.* 20 Ma (ref. 27) to compensate for a supercritically tapered mountain belt^26^.

Slab rollback orogeny also offers a solution for the current seismicity pattern where focal mechanisms of crustal events imply the occurrence of extensional forces at work beneath the Molasse Basin and within the Alps^35^,^57^, and it is consistent with the occurrence of normal faulting within the foreland plate. In addition, the slab load forces have been capable of compensating the buoyancy of the *ca.* 30-km-thick crustal root^31^,^33^, thereby keeping the Alpine topography at constant, but relatively low elevations during the past Millions of years.

In conclusion, a rollback mechanism yields an orogeny/foreland basin ensemble where subsidence and thrusting are partly decoupled at the scale of the orogeny. Such a model is capable of reconciling previously conflicting stratigraphic, palaeotopographic, seismic and plate tectonic observations in the Central Alps and the Molasse Basin. These mechanisms explain the formation of the Alps, and also of the Apennines^60^, through the delamination and accretion of crustal rocks from the subducting plate. Because the subsidence history has been partly decoupled from the build up of topographic loads, the Molasse Basin should not be considered as a classical foreland basin. In contrast, mountain belts that have evolved in a convergent plate tectonic regime where the subducting foreland plate advances towards the plate boundary offer more suitable conditions for relating orogenic growth with the development of a foreland basin. The plates underlying these basins will be deflected in response to horizontal compression forces and topographic loads, both of which act to downwarp the plate thereby contributing to the formation of a vertical subduction load force and a peripherial depression. The Siwalik foreland basin^61^, situated to the south of the Himalayas orogeny, and the Chaco foreland basin on the eastern margin of the Andes^62^ represent basins that have evolved under such conditions. We propose that these basins are more suited to serve as examples of a classical foreland basin as they have evolved in a convergent plate tectonic regime where orogeny and foreland plate deflection are closely linked.

## Methods

### Compilation of stratigraphic data from the Molasse Basin

We synthesize along a representative transect through the Central Alps published information about the history of crustal accretion of the Alps, the development of the adjacent Molasse foreland basin to the north and the evolution of the surface topography and sediment discharge between *ca.* 35 Ma and the present. For the Molasse Basin, estimates of the depth of the maximum deflection between 30 and 13 Ma (ref. 15) are based on compacted thicknesses of sections where magnetopolarity chronologies^7^,^8^,^9^,^10^,^11^ yielded a temporal resolution of *ca.* 500 Ka. Erosional recycling of previously deposited foreland material precludes a precise reconstruction of the subsidence history since 5–10 Ma when uplift and erosion started. Here fission track ages of detrital material encountered in deep Molasse drillings yielded constraints on the time and the amount of erosional recycling, yet with large uncertainties on both variables^41^,^42^. Flysch sequences that are 35–30 Ma old are *ca.* 1,000-m thick and are deposited close to the storm weather wave base^12^. Offshore marls that are 35 Ma old contain nearly 98% of planktonic foraminifera, which is consistent with an environment of nearly 1,000–1,300 m (up to 1,800 m) water depth^63^. Sediment discharge data for the Central Alps are based on sediment budgets of circum-Alpine basins^39^. Uncertainties of up to 50% in the data set include errors in the assignment of ages of the deposits and unknown thicknesses of sections that are buried beneath the Alpine nappes. Limited information about the thicknesses of sections that were eroded on thrusting of the proximal Molasse deposits and poor constraints on spatial differences in post-depositional compaction of Molasse sediments additionally contributed to substantial uncertainties in the sediment budgets^39^. The location of the orogeny front and the distal basin margin at different times were compiled from the literature^1^,^15^,^36^,^37^.

### Estimates of paleoaltimetries in the Alps

Reconstructions of the paleoalitmetry of the Alps have been accomplished through the use of empirical relationships between spacing of alluvial fans, orogen width and morphometric properties of stream channels feeding the fans^43^,^51^. According to these concepts, steepnesses and curvatures of longitudinal stream profiles can be used to reconstruct the topographic evolution of a mountain belt through time. It has been shown that the spacing of alluvial fans *F* scales with orogen widths *W* (ref. 45):


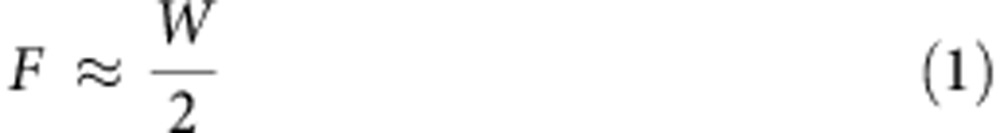


Alternatively, the width *W* is nearly equivalent to the length *W*≦*L* along which streams have dissected into mountain belt from the orogen margin to the drainage divide in the rear of the mountain belt. The length *L* of this reach scales with the drainage area *A* through a power law relationship^64^,^65^:


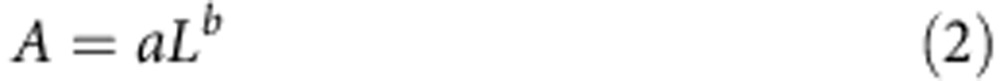


where *a* and *b* are constants that depend on the shape of the drainage basin^64^,^65^. While the values of 0.5 and 1.77 have been assigned to the variables *a* and *b* based on the morphometric properties of current streams in the Central Swiss Alps^43^ (which we have applied here), other compilations yielded values close to 2 (1.94) for the exponent *b*^51^,^66^. Finally, in tectonically active mountain belts where streams are bedrock channelled, the gradients of these streams can be related to the upstream accumulation area through^64^:


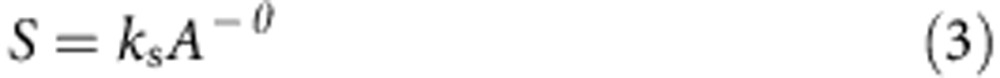


where *S* is the channel gradient, *A* is the upstream accumulation area, *k*_s_ is the channel steepness and *θ* is the channel concavity. Therefore, the total fluvial relief *H*, used here as proxy for the elevation of the Alpine drainage divides relative to the depocentres, can be calculated through the integration of slopes/elevations along the channel lengths from the fan depocentres to the drainage divides^67^. For paleotopogaphy estimates at 15 Ma, independent constraints are provided by oxygen isotope data established on dated fault gauges in the Alps^53^.

Concavities *θ* of streams in mountain belts and particularly in the Swiss Alps range between *ca.* 0.75 and 0.45 depending on whether or not the landscape has been substantially perturbed by glacial processes^50^. While we cannot fully exclude the occurrence of Alpine glaciers during the Oligocene and Miocene, the record of fossiliferous plants suggests that climate at that time was most likely warmer than at present^68^,^69^, which implies that the glacial imprint on the Oligo/Miocene landscape of the Alps was presumably much less than at present. If it were, then glacial landforms were most likely restricted to the headwaters. Accordingly, following the arguments presented in previously published articles, a reference concavity *θ* of 0.45 (ref. 48) and channel steepnesses *k*_s_ of 200±100 m were applied for the Alpine rivers^46^,^47^,^48^,^49^,^50^,^51^.

### Reconstruction of tectonic evolution of the Alps

The post-collisional plate tectonic evolution of the Central Alps is illustrated as conceptual model for different time steps when changes in the basin geometry and the depositional style in the Molasse Basin are well constrained. These are as follows: (a) 35 Ma when Flysch sedimentation prevailed, (b) 30 Ma when the foreland basin became overfilled, (c) 25 Ma when deposition of fluvial deposits in a overfilled basin prevailed, (d) 20 Ma, which marks the transgression of the UMM and (e) 10 Ma when folding of the Jura Mountains started. Our reconstruction considers the following: (i) isodynamic force equilibrium between loads and buoyancy for a lithosphere floating on asthenosphere, (ii) the conservation of mass, which is adapted here through crustal mass and line balancing and (iii) changes in the flexural rigidity of the rollback subducting plate. Lateral shifts of lithotectonic units caused by the anticlockwise rotation of the Adriatic plate are not considered in these diagrams. While these processes have been used to model the plate deflection in the rear of the Alps, they cannot be invoked to explain the increase in the basin's amplitude after 20 Ma (ref. 15). Our area-balanced reconstruction starts from the present-day plate tectonic architecture across the Central Alps (Fig. 1), which has originally been based on seismic reflection and refraction profiles^14^,^18^, and which has been improved based on results of high-resolution local earthquake^70^ and teleseismic surveys^18^,^19^,^20^. On the northern side, shifts in the orogen front are based on restorations of the proximal foreland basin margin adjacent to the Alps, illustrated here with the variables Δ_Ν1_ and Δ_Ν2_ (Figs 2 and 5). Accordingly, for the time between *ca.* 30 and 10 Ma, the cumulative length of subduction (160 km)^19^ corresponds to the total amount of shortening on the northern and southern sides of the Alps (120 km)^14^ plus the 40-km-northward shift of the orogeny front as inferred from Fig. 2. Finally, the chronology of intracrustal delamination has been adapted from the literature^14^,^18^.

## Additional information

**How to cite this article:** Schlunegger, F. & Kissling, E. Slab rollback orogeny in the Alps and evolution of the Swiss Molasse basin. *Nat. Commun.* 6:8605 doi: 10.1038/ncomms9605 (2015).
